# Gene expression profiling of liver metastases and tumour invasion in pancreatic cancer using an orthotopic SCID mouse model

**DOI:** 10.1038/sj.bjc.6604031

**Published:** 2007-10-16

**Authors:** M Niedergethmann, F Alves, J K Neff, B Heidrich, N Aramin, L Li, C Pilarsky, R Grützmann, H Allgayer, S Post, N Gretz

**Affiliations:** 1 Faculty of Medicine Mannheim, Department of Surgery, University-Hospital Mannheim, University of Heidelberg, Mannheim 68135, Germany; 2 Department of Hematology and Oncology, University-Hospital Göttingen, Göttingen 37075, Germany; 3 Department of Medical Research, Faculty of Medicine Mannheim, Medical Research Centre, University of Heidelberg, Mannheim 68135, Germany; 4 Department of Surgery, University-Hospital Dresden, Dresden 01307, Germany; 5 Faculty of Medicine Mannheim, Department of Experimental Surgery and Molecular Oncology of Solid Tumors (DKFZ), University of Heidelberg, Mannheim 68135, Germany

**Keywords:** pancreatic cancer, gene expression profiling, overrepresentation analysis, apoptosis, angiogenesis, cell migration

## Abstract

The prognosis of pancreatic adenocarcinoma is affected by early metastases and local tumour invasion beyond surgical margins. Gene expression profiling in pancreatic cancer tissue is complicated due to the high amount of RNAses being present in human tissue and that of suitable models. In order to demonstrate early metastases, the models should take into account the anatomical environment of the tumour. Using the orthotopic transplantation of pancreatic tumour cells in SCID (severe combined immunodeficiency) mice, these interactions are taken into consideration. In order to identify genes associated with local tumour invasion and metastases in ductal pancreatic cancer, we investigated a human pancreatic tumour cell line derived from an orthopic pancreatic tumour model in SCID mice. Differential gene expression was performed on the basis of microarray technique. The human MiaPaca-2 cell line was implanted orthotopically in SCID mice. Transcriptional profiling was performed on fresh frozen tissue derived from the primary tumour, the tumour invasion front and the liver metastases. Differentially expressed genes were identified using statistical analyses, and were validated with external databases and with immunohistochemistry. A total of 1066 of 14 500 genes were significantly differentially expressed. Comparing the primary tumour with the tumour invasion front, there were 614 statistically significant up- and 348 downregulated genes. Twenty-five statistically significant up- and 181 downregulated genes were identified comparing the liver metastases with the primary tumour. Eight genes (PAI-1, BNIP3l, VEGF, NSE, RGS4, HSP27, GADD45A, PTPN14) were chosen and validated in a semi-quantitative immunohistochemical analysis, which revealed a positive correlation to the array data. Overrepresentation analyses revealed a total of 66 significantly regulated pathways associated with cell proliferation, cell stress, cell communication metabolic and cytokine function. In conclusion, model marker genes for local invasion and liver metastases can be identified using transcriptional profiling in the SCID mouse. Overrepresentation analysis secures a good and fast overview about the significantly regulated genes and can assign genes to certain pathways. These marker genes can be related to the apoptotic cascade, angiogenesis and cell interaction.

Although the treatment of various types of cancer has improved over the past few years, the prognosis of pancreatic ductal adenocarcinoma (PDAC) remains poor. Pancreatic cancer is the fifth leading cause of cancer deaths in the United States (Jemal *et al*, 2003), and shows a 5-year survival rate of less than 5%. The poor prognosis can be explained by high rate of local recurrences and distant metastases after surgery, as well as frequent diagnosis being made at late stages of disease. This limits the role of surgery as a curative modality. Despite all modern diagnostic tools early detection of PDAC, even in an extended disease has been difficult up to now (Eskelinen and Haglund, 1999). Furthermore, lymphatic or distant metastases as well as unknown tumour infiltration into large vessels is frequently observed in potentially curative resections (Hartel *et al*, 2002; Richter *et al*, 2003), further limiting the surgical therapeutic options (Trede *et al*, 2001). In the past few years, a number of cancer-related genes could be identified in pancreatic cancer, the most common of which are K-ras, DPC4, p53 and p16 (Almoguera *et al*, 1988; Hahn *et al*, 1996; Hruban *et al*, 1998; Slebos *et al*, 2000). However, the published array data are limited to the differential gene expression between normal pancreatic tissue and PDAC, and does not address important aspects such as progression and metastasis (Pilarsky *et al*, 2004). The genome is very complex and most of the molecular changes causing pancreatic cancer are still unknown. Most importantly there is a lack of knowledge about the molecular components of early progression and metastases, which are two main reasons for the overall poor prognosis. In order to reflect early metastases, there is a demand for models representing the anatomical environment of the tumour. Using the orthotopic transplantation of pancreatic tumour cells into SCID (severe combined immunodeficiency) mice, these interactions are taken into account (Alves *et al*, 2001). In order to identify genes associated with local tumour invasion and metastases in PDAC, we examined pancreatic tumour cells derived from an orthotopic pancreatic tumour model in SCID mice regarding their potential to establish metastases and to infiltrate duodenum. Gene expression profiling was performed on the basis of microarray technique. The biological functions of the differentially expressed genes were analysed by means of pathway analysis, and the changes of gene expression at the level of biological pathways or co-regulated gene sets were evaluated. Furthermore, we validated genes of this set using immunohistochemistry in order to prove the appropriateness of our approach.

## MATERIALS AND METHODS

### MiaPaca-2 cell line

The human pancreatic ductal adenocarcinoma cell line Mia PaCa-2 (established by Yunis *et al*, 1977) was provided by Kalthoff *et al* (1993) (Kiel, Germany). The cells were cultured in RPMI 1640, which was supplemented with penicillin (50 IU ml^−1^), streptomycin (50 *μ*g ml^−1^), L-glutamine (2 mM) (Gibco BRL, Eggenstein, Germany) and 10% foetal calf serum (PAN, Aidenbach, Germany), at 37°C in a humidified atmosphere of 5% CO_2_. The cultured cells were gained by short trypsinisation in 0.25% trypsin–EDTA solution (Gibco, Karlsruhe, Germany) from semiconfluent culture dishes and washed several times. Shortly before implantation, the cells were placed into sterile phosphate-buffered saline (PBS). In addition the MiaPaca cells were fixed in Hank's balanced salt solution containing 4% formaldehyde and were processed for paraffin embedding.

### SCID mouse model

The SCID mouse model, which presents an orthotopic and metastatic model, was established by Alves *et al* (2001). Severe combined immunodeficiency mice (age 24–28 weeks; strain C.B-17/Ztm-scid of both sexes; weight 24–26, 4 g (female) and 26, 3–32, 4 g (male)) were used for the tumour implantation. By measuring serum immunoglobulin levels by Ouchterlony test and using an enzyme-linked immunosorbent assay for immunoglobulin, leaky SCID mice (immunoglobulin>10 *μ*g ml^−1^) were detected and excluded from further experiments. All animals were maintained in a sterile environment in special cages with filter hoods in a scantainer (Scanbur, Koge, Denmark) on a daily 12-h light/dark cycle. Cages, bedding and water were autoclaved and the chow was sterilised by gamma radiation. All manipulations were conducted under aseptic conditions using a laminar flow hood. In accordance with guidelines for animal welfare, all experiments performed were approved by the administration of Niedersachsen (Lower Saxony, Germany).

### Tumour implantation

Under general anesthesia using a mixture of ketamine (75–100mg kg^−1^) and xylazine (15–20 mg kg^−1^), the orthotopic transplantation was performed intraperitoneally. A median laparotomy was performed, approximately 1 cm in length, peritoneum was opened, the pancreas was carefully exposed by applying gentle traction to the stomach. Aliquots of 1 × 10^6^ MiaPaca-2 cells in a volume of 15 *μ*l PBS were injected with an insulin syringe, 29 gauge (Becton Dickinson, Heidelberg, Germany), very slowly into the proximal part of the pancreas. The implanted cells were visible as an infiltration in the pancreatic tissue. The needle was slowly withdrawn after 1 min. After returning the pancreas to the abdominal cavity, the incision was closed in two layers using a continuous Vicryl suture (Metric 1.5; Ethicon, Norderstedt, Germany) for the peritoneum and an interrupted suture for the skin. The procedure was tolerated well by all the animals. After the implantation, the mice were inspected daily for body weight loss, general condition and tumour formation in the peritoneal cavity.

### Termination of the trial

In case of a rapid tumour progression, the trial was terminated (usually 39–60 days) and the general condition of the animals (defined in terms of the condition of the coat, nutrition and behaviour) was assessed daily. The mice were killed after an average of 56 days. Autopsies were performed and the abdominal and the thoracic cavities were examined systematically for the presence of metastases. All findings and complications, such as ascites, level of invasion into the surrounding tissue, the size, number and the location of tumours were recorded. The pancreatic tumour mass, including the attached organs, metastases, suspicious nodular formations, lung, liver, spleen, kidneys, adrenal glands, diaphragm, various parts of the intestine, bladder and male and female reproductive organs were excised, placed in phosphate-buffered 4% formalin for 16 h at room temperature and embedded in paraffin. In addition, three different specimens from the primary tumour, the tumour invasion front and the liver metastases were dissected, snap frozen and stored at −80°C for further analyses.

### RNA preparation and array hybridisation

The RNA was isolated using TRIZOL-Reagent (Invitrogen, Carlsbad, CA, USA). After measuring the concentration of RNA and approving for purity by Agilent Bioanalyzer 2100, the RNA was transferred into cDNA using Superscript II (Invitrogen, Carlsbad, CA, USA). In a second step, the cDNA was treated by RNA-transcript-labelling kit (Enzo Diagnostics, Farmingdale, NY, USA) in order to obtain biotin-labelled RNA. Hybridisation and detection of the labelled RNA on the U133 Affymetrix GeneChip were performed according to the instructions of Affymetrix.

### Design of the chip experiment and the bioinformatical analysis

Gene expression profiling was performed using arrays of HG-U133A type from Affymetrix. The 22 000 probe sets represent 18 400 transcripts and 14 500 genes. In total nine arrays, three containing primary tumour material, three containing material of the tumour invasion front and three containing liver metastases material were hybridised. Only probe sets unique to single transcripts with the Affymetrix extension _at, or those unique to common parts among transcripts from the same gene with extension _s_at were taken into consideration. Differential gene expression was analysed based on loglinear mixed model ANOVA (Hsieh *et al*, 2003; Roy, 2007), using a commercial software package Micro Array Solution, version 1.0, from SAS (SAS Institute, Cary, NC, USA). The experimental group and probe were considered to be with fixed effects and the chipid random. A false positive rate of *a*=0.05 with Bonferroni correction (according to a *P*-value slightly smaller than 0.000001) was taken as the level of significance. The results were presented in lists of significantly regulated genes. The three different types of tissue, the primary tumour, the tumour invasion front and the liver metastases, were compared with each other.

### Pathway analysis

The overrepresentation analysis (ORA) is a microarray data analysis that uses predefined gene sets to identify a significant overrepresentation of genes in data sets (Subramanian *et al*, 2005; Manoli *et al*, 2006). Pathways belonging to various cell functions such as cell cycle or apoptosis were obtained from public external databases (KEGG: www.kegg.com, Superarray: www.superarray.com). A Fisher's exact test was performed to detect the significantly regulated pathways.

### Immunohistochemical staining

Based on the list of significantly regulated genes (Table 1), we selected the following eight genes for further immunohistochemical validation: BNIP3L, regulator of G protein signalling 4 (RGS4), plasminogen activator inhibitor 1 (PAI-1), GADD45A, PTPN14, vascular endothelial growth factor (VEGF), neuron-specific enolase (NSE) and HSP27. Sections of formalin-fixed, paraffin-embedded tumour tissue with a thickness of 3 *μ*m were deparaffinised and dehydrated. The slides were pretreated in a microwave (900 W/15 min in citrate buffer at pH 6.0) and were treated for 5 min with hydrogen peroxide (3% hydrogen peroxide in methanol). They were then blocked with non-diluted serum for 30 min. Goat serum was used for PAI-1, GADD45A, PTPN14, VEGF, NSE and HSP27. For RGS4 staining we used rabbit serum. For the liver metastases specimen, we employed the avidin–biotin blockade due to the high amount of biotin being present. The primary antibodies were incubated at 4°C overnight. In order to stain the BNIP3L protein, we administered rabbit polyclonal anti-BNIP3L antibodies (dilution: 1 : 100, Abcam plc, Cambridge, England), and for the RGS4 we chose goat polyclonal anti-RGS4 antibodies (dilution 1 : 400, Santa Cruz Biotechnology Inc., Santa Cruz, CA, USA). K 562 cells were used as positive control for the BNIP3l antibody, and human brain tissue was used as positive control for the RGS4 antibody. Anti-PAI-1 antibodies are rabbit polyclonal antibodies (Abcam plc, Cambridge, England), and these were administered at a 1 : 50 dilution. Breast tissues, normal tissues and cancer tissues were used as positive control. We used the rabbit polyclonal anti-GADD45A antibodies at a 1 : 500 dilution (Santa Cruz Biotechnology Inc., Santa Cruz, CA, USA); the K 562 cells served as positive control. PTPN14 was detected with chicken polyclonal anti-PTPN14 antibodies (1 : 300; Biomol GmbH, Hamburg, Germany). As positive control we used pancreatic and breast tissue. The VEGF protein was stained with goat-polyclonal anti-VEGF antibodies (1 : 300, Santa Cruz Biotechnology Inc., Santa Cruz, CA, USA). Vein tissue was used as positive control. For NSE staining we chose rabbit polyclonal anti-NSE antibodies (1 : 300; Abcam plc, Cambridge, England) and pancreas tissue as positive control. And finally, for the protein HSP27 we selected rabbit polyclonal anti-HSP27 antibodies (1 : 250; Abcam plc, Cambridge, England). As positive control we stained breast and cervix normal and cancer tissues. The secondary antibodies were applied with polyclonal anti-rabbit antibodies, except for RGS4 and PTPN14 staining. For the latter two we used anti-goat and anti-chicken antibodies (Vector Laboratories Ltd., Peterborough, England) for 30 min at room temperature. All immunostainings were performed for 30 min at room temperature using the streptavidin–biotin method (Vector Laboratories Ltd., Peterborough, England). The stainings were detected by 3,
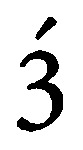
-diaminobenzidine. For the PAI-1 staining, however, we applied the NovaRed method (Vector Laboratories Ltd., Peterborough, England). The slides were counterstained with Mayer’s haematoxylin.

### Evaluation of protein expression

Semi-quantitative assessment of the immunhistochemistry staining was based on scoring the number of all positive cells per 200 × field, including carcinoma/metastasis/invasion front, and stromal cells (fibroblasts, macrophages, endothelial cells and lymphocytes). Normal pancreatic parenchyma was also screened for the presence of protein expression in acinar, ductal and endocrine cells. The tumour cell density was similar in all samples. In each slide three fields were evaluated. If there was no staining observed, the case was scored as ‘0’. If the tumour tissue consisted of less than 10% immunoreactive cells, the case was rated as ‘1’ (weak), while cases with 10–50% immunoreactive cells were scored as ‘2’ (moderate) and those with more than 50% immunoreactive cells were scored as ‘3’ (strong). The semi-quantitative assessment of all parameters was performed by two independent observers (Niedergethmann (M) and Neff (JK)).

## RESULTS

### Bioinformatical analysis

The array-based gene expression analysis revealed a total of 1066 statistically significant differentially expressed genes (*P*-value⩽10^−6.13^). Comparing the gene expression profile of the primary tumour with the tumour invasion, there were 614 genes upregulated and 348 genes downregulated. In comparison to the primary tumour, there were 25 genes overexpressed and 181 genes underexpressed in the liver metastases. Furthermore, we found 73 genes upregulated and 37 genes downregulated comparing liver metastases with tumour invasion front. We noted a homogenous distribution of significantly regulated genes in all specimens. We selected eight significantly regulated genes based on their high *P*-value and a greater log 2 fold change (>1). In liver metastases and the tumour invasion front, these genes revealed a statistically significant downregulation compared with the primary tumour. We compared these genes with the data from the current literature: *BNIP3L, RGS4, PAI-1, GADD45A, PTPN14, VEGF, NSE* and *HSP27* (Table 1). Based on the comparison with external databases and using immunohistochemistry, the differential expression of these genes was validated.

### Pathway analysis

The ORA revealed a total of 66 significantly regulated pathways. The comparison of the primary tumour with the tumour invasion front showed 30 significantly regulated pathways. The comparison of the metastases with the tumour invasion front demonstrated 13 and that of the metastases with the primary tumour revealed 23 significantly regulated pathways. In Table 2 all pathways, which could be associated with important cell interactions in pancreatic cancer are summarised. Pathways linking the primary tumour with the tumour invasion front or with the liver metastases are mainly involved in cell proliferation, cell stress and cell communication. Pathways between primary tumour and tumour invasion front are associated with cytokine function. Those linking primary tumour with liver metastases are metabolic pathways.

### Comparison with published data

In order to interpret our results in a general context, we analysed data that have already been published on this topic over the past few years (Han *et al*, 2002; Iacobuzio-Donahue *et al*, 2003a; Grützmann *et al*, 2004). We found that 75 of our genes had already been reported by other institutions, which analysed PDAC with microarrays. For clarity, only those genes with *P*-values lower than 10^−6.13^ are presented in Table 3. Regarding the significantly regulated pathways, however, we found out that only those involved in cell proliferation, cell stress and cell communication have already been described before (Grützmann *et al*, 2004; Table 2, section A), whereas those being associated with cytokine or metabolic function (Table 2, sections B and C) have not been described yet.

### Immunohistochemical staining

**Figure 1:**
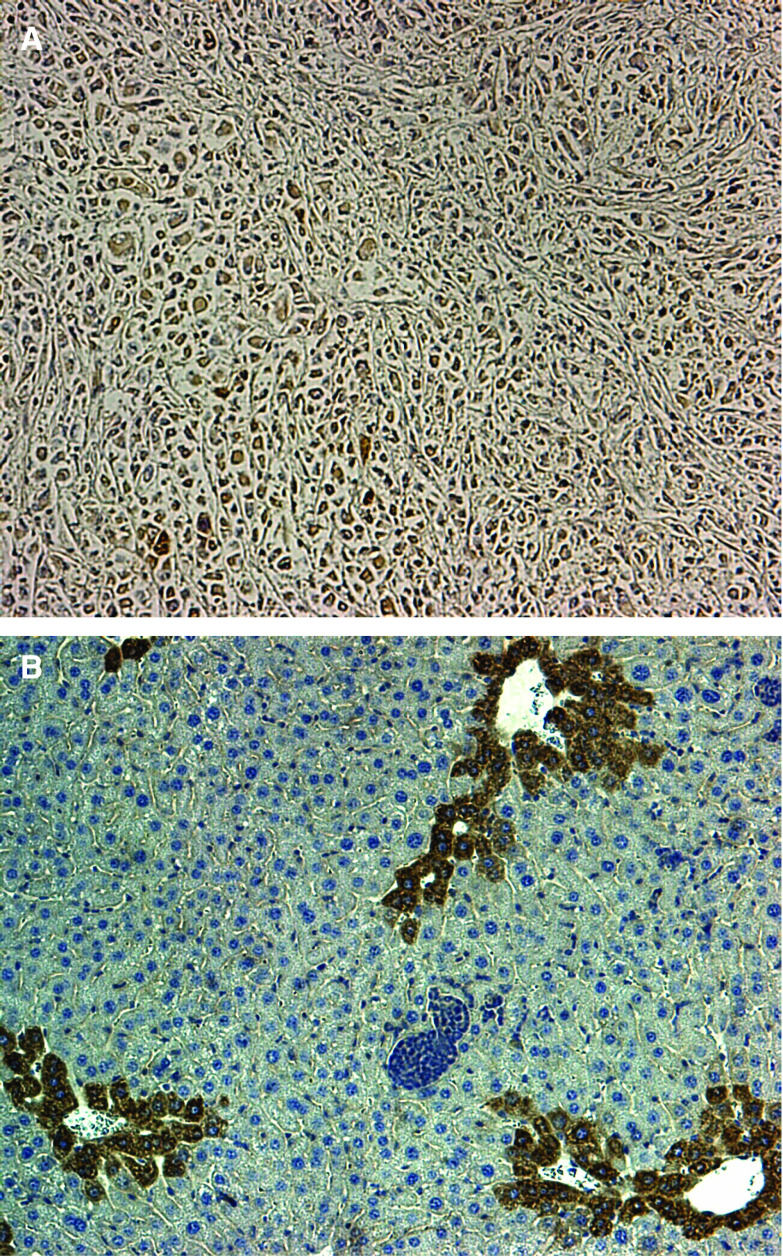
**(A, B)** Immunohistochemistry of HSP27 showing equal staining intensity in the primary lesion (**A**) and the liver metastasis (**B**), since the gene expression was as well equal in the array ( × 200 magnification).

**Figure 2:**
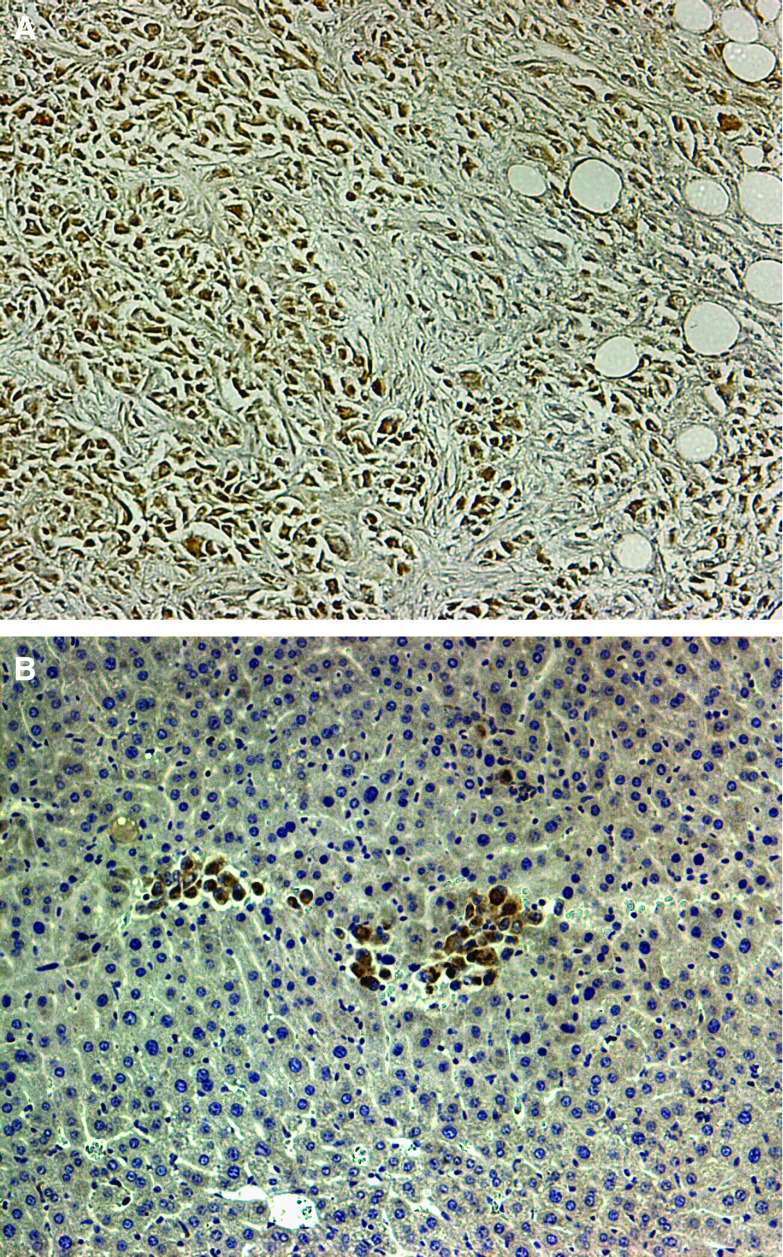
**(A, B)** Immunohistochemistry of BNIP3L of the primary tumour (**A**) and of the liver metastasis (**B**) with a weaker staining ( × 200 magnification).

**Figure 3:**
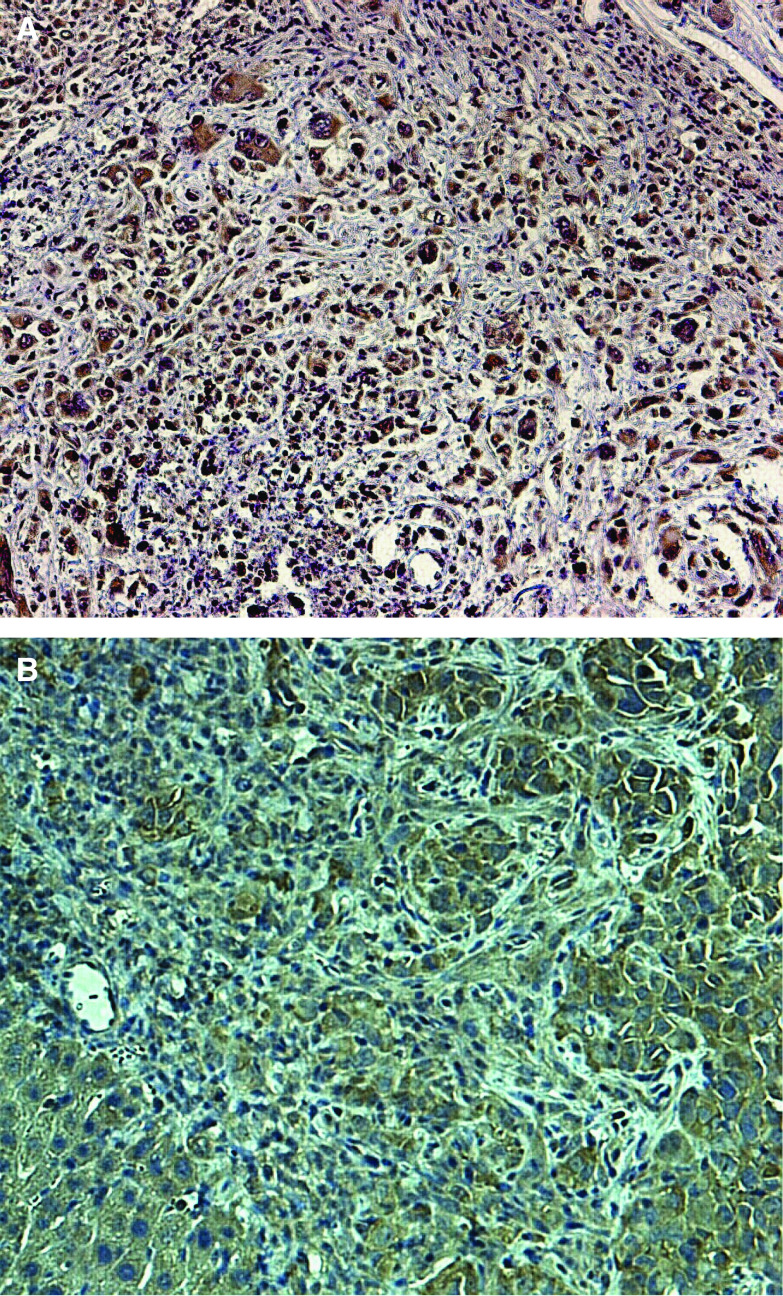
**(A** and **B)** Immunohistochemistry of PTPN14 with a strong staining in the primary lesions (**A**) compared with a weak staining in the liver metastasis reflecting equal array results ( × 200 magnification).

For all genes selected, the differential expression of the resulting protein could be confirmed by immunohistochemical staining. For all chosen genes downregulated in liver metastases and invasion front in comparison to the primary tumour, such as VEGF, BNIP3l and HSP27, we found a stronger cytoplasmatic and nuclear immunoreactivity in the primary tumour in comparison with liver metastases/tumour invasion front (examples shown in Figures 1, 2 and 3). (All protein stainings are detected in cancer cells. Vascular endothelial growth factor staining was also observed in endothelial cells. Antibodies to proteins such as VEGF, PAI-1, HSP27, NSE, PTPN14 and BNIP3L showed a strong immunoreactivity in our study.) The immunoreactivity of the BNIP3L and PTPN14 staining was strong in the primary tumour (score 3), whereas weak staining was observed in the liver metastases (score 1). The immunohistochemical staining of GADD45A and RGS4 was weak in all localisations. The results of array data in comparison to immunohistochemistry scoring are represented in Table 4 and in Figures 1, 2 and 3.

## DISCUSSION

Since the establishment of the human genome project, sequence information of the entire human genome has become available. The human genome can be used for global analyses of gene expression in the form of DNA microarrays. Over the past few years, gene expression profiling has been applied to a number of tumours such as breast, colon, prostate, oesophagus and stomach tumours (Grützmann *et al*, 2004). Several studies using different methods have elucidated gene expression changes in PDAC (Iacobuzio-Donahue *et al*, 2003a; Grützmann *et al*, 2004). The use of an orthotopic animal model has recently been described by Nakamura *et al* (2007) as an appropriate method of reflecting the metastasising cascade of tumours in human beings. The purpose of this study was to identify genes associated with metastases and/or local tumour invasion in PDAC. Tumour lesions could easily be obtained even in cases of liver metastases or the tumour invasion front. Therefore a microdissection was not necessary in our model. Using the well-established SCID mouse model we could gain a good quality of RNA from the primary tumour, the tumour invasion site and the liver metastases.

The gene expression analysis revealed a total of 1066 significantly differentially regulated genes. Comparing the tissue from the primary tumour with the tumour invasion front, we observed 614 upregulated and 348 downregulated genes. Twenty-five upregulated genes and 181 downregulated genes could be identified comparing the metastases with the primary tumour. A comparison of the metastases with the tumour invasion revealed 73 upregulated and 37 downregulated genes. Remarkably, we could find less upregulated genes in the liver metastases than in the primary tumour, indicating a possible loss of regulating and tumour suppressor genes. The same observation has been made by other groups (Li *et al*, 2004; Zucchi *et al*, 2004). Zucchi *et al* (2004) hypothesised that the downregulation of a set of genes may be the basic mechanism of cancer formation, whereas the upregulation may characterise and possibly control the state of evolution of individual cancers.

A number of these genes have already been reported by other authors. We noticed that 75 genes that have already been reported were also significantly regulated in our study (Han *et al*, 2002; Iacobuzio-Donahue *et al*, 2003a; Grützmann *et al*, 2004; Table 3). However, we found additional genes that have not yet been found to be differentially expressed in pancreatic cancer, but have been identified within other malignancies. The largest numbers of the significantly regulated genes are those that have not yet been associated with any type of carcinoma. Furthermore, using our metastasising model we could describe for the first time genes such as PAI-1 (SERPINE 1) and HSP27 showing a downregulation in the liver metastases as well as in the tumour invasion front (Tables 1 and 4).

In order to define the biological function of the genes and for purposes of validation, we analysed the changes in the gene expression at the level of biological pathways or co-regulated gene sets. Overrepresentation analysis is one way of handling the large amount of data and obtaining an adequate and efficient overview of a microarray data set, which in turn allows to obtain biologically valid data. Using the ORA we observed 30 significant pathways comparing the primary tumour with the tumour invasion front, 13 pathways comparing the liver metastases with the tumour invasion front and 23 pathways comparing the primary tumour with liver metastases. We summarised relevant pathways for pancreatic cancer and assigned them to certain cell functions (such as cell proliferation) (Table 2). In pancreatic cancer pathways playing a role in cell proliferation, cell stress and cell communication have already being described before (Grützmann *et al*, 2004). Using our metastasising model, we could detect specific pathways being possibly linked with liver metastases or the local tumour invasion front. Those, which might be associated with liver metastases are mainly involved in metabolic function and reflect the scope of liver synthesis. On the other hand, those, which might be linked with the tumour invasion front consist of cytokine function and therefore present the typical pancreatic cancer peritumoral inflammation. This implicates further cross-validation and confirmation with for instance intraoperatively extracted tissue in a series with patient specimen.

For all three tissue specimen, only the so-called Cosmic Cancer and the tgf-beta-signalling pathway were significantly expressed. In the Cosmic Cancer pathway, multiple somatic mutations in cancers are comprised based on a study by Futreal *et al* (2004). Additionally we found seven pathways, such as nitric oxide and WNT-signalling pathway, which were significantly regulated in primary tumour in comparison with the tumour invasion front and the liver metastases. Comparing the primary tumour with the tumour invasion front, we found other significantly regulated pathways such as growth factor and common cytokines, which play an important role in cell growth and differentiation (Table 2). Not surprisingly, the pathway growth factor is significantly regulated, as it is a marker pathway for a selective growth advantage (Westphal and Kalthoff, 2003). Human pancreatic cancer cells are reported to produce multiple growth factors like insulin-like growth factor (IGF) and its receptors (IGF-1R and IGF-2 receptor), epidermal growth factor (EGF), transforming growth factor-alpha (TGF-alpha), transforming growth factor-beta (TGF-beta), fibroblast growth factor (FGF), vascular endothelial growth factors (VEGFs) and their receptors (Westphal and Kalthoff, 2003). Remarkably, for the link between the primary tumour and liver metastases, apoptosis-related pathways like apoptosis-bcl2 and map kinase active transcription factor are significantly regulated (Table 2). Apoptosis and the MAP kinase are known to play a pivotal role in pancreatic cancer (Westphal and Kalthoff, 2003). Subsuming ORA is able to identify specific pathways correlating with a specific biological behaviour.

Furthermore, we defined a panel of eight significantly regulated genes showing high *P*-values and high log 2 fold changes. These were compared with the data in the current literature. This panel consists of *VEGF*, *NSE*, *HSP27*, *BNIP3L*, *PAI-1*, *GADD45A*, *PTNPN14 and RGS4* (Table 1), and these genes are validated by immunohistochemistry (shown exemplary in Figures 1, 2 and 3).

Vascular endothelial growth factor is a marker of prognostic relevance and a predictor for an early recurrence after curative resection of ductal adenocarcinoma of the pancreas (Niedergethmann *et al*, 2002). We found VEGF to be downregulated in liver metastases (Table 1), underlining its key role in tumour-cell migration and haematogenous spread in primary lesions. Similar to our setting, Fukuhara *et al* (2005) showed that the VEGF expression is initially reduced in liver metastases. We have already demonstrated a correlation between the intensity of VEGF expression in PDAC and the biological behaviour in terms of early recurrence and poor survival after R0 resection in our earlier work (Niedergethmann *et al*, 2002). Immunohistological staining showed a high immunoreactivity with a score of 3 in primary tumours, whereas in the liver metastases, VEGF expression rate was lower with a score of 2. The tumour invasion front, however, displayed a staining with a score of 2.8. The results of immunohistochemistry are comparable to those of the array data. It can therefore be postulated that underexpression and low immunoreactivity in the liver metastases in comparison with the primary tumours reflect the pivotal role of angiogenesis in primary lesions.

Neuron-specific enolase, the glycolytic isoenzyme of the enolase gamma–gamma dimer, is a specific marker for the diffuse neuroendocrine system and derivate tumours. It can be considered as a reliable marker in the differential diagnosis between endocrine and neuroendocrine neoplasias (D’Alessandro *et al*, 1992). Its concentration is elevated in plasma in certain neoplasias including paediatric neuroblastoma and small-cell lung cancer (Massaron *et al*, 1998; Song *et al*, 2004). In anaplastic carcinoma of the pancreas (Sawai *et al*, 2005) and in solid-cystic (papillary cystic) tumour of the pancreas, the protein NSE is expressed (Chott *et al*, 1987). In the immunohistochemical staining, NSE showed stronger staining (score 3) in primary tumour than in liver metastases (score 2). The tumour invasion front revealed also a lower staining in comparison with the primary tumour, with a score of 2.4, analogous to the results from the array data (Table 4).

The heat shock 27-kDa protein 1 (HSP27) belongs to a larger group of polypeptides, the stress proteins, and its reduced expression, as in our setting, is an early marker of poor prognosis. It is for instance useful in identifying aggressive biological behaviour in oral squamous-cell carcinoma (Lo Muzio *et al*, 2006). Recently, HSP27 has been described as a potential marker for pancreatic cancer (Melle *et al*, 2007). Considering the staining results there is no difference between the primary tumour, the liver metastases and the tumour invasion front. All tissues present a staining with a score >2. Therefore, HSP27 is not specific enough to reveal differences in the progression of pancreatic cancer.

BNIP3L is a member of the BCL-2 family, which are critical regulators of apoptosis by either inhibiting or promoting cell death. BNIP3L protein expression is downregulated in lung cancer (Sun *et al*, 2004). Similar to lung cancer we found BNIP3L to be downregulated in metastases and tumour invasion front in our model, underlining its pivotal role in apoptosis. Han *et al* (2002) have already described the expression of BNIP3l in PDAC. The results of the staining revealed the highest staining score of 3 for BNIP3l in the primary tumour. In addition there is a decrease in immunoreactivity comparing the tumour invasion front (score 2.2) with the liver metastases (score 1).

Plasminogen activator inhibitor 1 is a member of the serpine family of serine protease inhibitors, and is one of the key regulators of tumour invasion and metastases (Inoue *et al*, 2005; Beyer *et al*, 2006). In oral squamous-cell carcinoma PAI-1 is a novel potential marker of initial invasion (Lindberg *et al*, 2006). As previously reported by our group (Niedergethmann *et al*, 2004), similar to plasminogen and cathepsin B/C and their inhibitors, the serine proteases are of paramount importance in local invasion and metastases in PDAC. Takeuchi *et al* (1993) could already demonstrate a high PAI-1 expression in human pancreatic carcinomas in an immunohistochemical study. There are data advocating that the uPA/uPAR/PAI-1 system is activated in advanced pancreatic cancer and may account for the tumour's aggressive behaviour (Smith *et al*, 2007). We observed a high expression of PAI-1 in the primary tumour, the tumour invasion front and the metastases. The staining showed a slight decrease of immunoreactivity when comparing the primary tumour (score 3) with the tumour invasion front (score 2.4) and metastases (score 2).

The growth arrest and DNA damage-inducible gene (GADD45A) is a cell-cycle protein. It is an effective indicator of poor prognosis in solid tumours such as lung cancer. In non-small cell lung cancer, the downregulation of GADD45A is regarded as an important key in the differentiation pathway (Higashi *et al*, 2006). Hollander *et al* (1999) could demonstrate that GADD45A is a component of the p53 pathway, contributing to the maintenance of genomic stability. Both p53 and GADD45A are highly expressed in human pancreatic cancer and may be associated with pivotal biological features of pancreatic cancer (Dong *et al*, 2005). Downregulation of GADD45A, as in our setting, reduces proliferation and induces apoptosis in pancreatic cancer cells (Schneider *et al*, 2006). GADD45A revealed a weak staining in all tissues with a score of 1.

PTPN14 is a member of the protein tyrosine phosphatase family, which is increased in many tumours, indicating that a complex dysregulation in the balance of tyrosine phosphorylation could be responsible for major alterations in various cellular processes (Freiss and Vignon, 2004). Wang *et al* (2004) performed a mutational analysis of the tyrosine phosphatase gene superfamily in human cancers, and identified 83 somatic mutations in six protein-tyrosine phosphatases one of which is PTPN14. PTPN14 demonstrated a high immunoreactivity score of 2.4 in the primary tumour, whereas there was no decrease observed in the tumour invasion front (score 2.4). In liver metastases we observed a score of 1, confirming the hybridisation data described.

Regulator of G protein signalling 4 is regarded as a novel inhibitor of tubulogenesis; it inhibits mitogen-activated protein kinases and VEGF signalling (Albig and Schiemann, 2005). Due to its downregulation in our model, one could speculate that its inhibitory role is lost in PDAC. The immunohistochemical staining was weak. In the primary tumour it showed a score of 1, in the tumour invasion front a score of 1.2 and no immunoreactivity was found in the liver metastases.

Using the SCID mouse model we could obtain a convincing panel of genes associated with local invasion and metastases. Beside current diagnostic work-up for PDAC, this gene and pathway panel may deliver additional information about tumours, which have already spread beyond surgical margins. This implicates further validation in a series of human patient specimen. Since resectability cannot be sufficiently predicted using the conventional diagnostic measures, this additional information might be helpful in planning the appropriate treatment. On the other hand, local involvement of vessels and surrounding organs is still a diagnostic problem. Having additional staging details by possible biomarkers physicians can be provided with information for planning the further procedure. Furthermore, panels such as that of ours are of importance in understanding the biological behaviour of PDAC.

In conclusion, microarray-based transcript analyses have broadened our understanding of tumour biology. Based on metastasising models such as the presented SCID mouse model, biological behaviour can be studied and analysed. These data may influence future diagnostic and therapeutic strategies for the treatment of PDAC. With the rapid progress made in the handling and analysis of microarrays, it can be assumed that more insight will soon be gained into the fundamental changes occurring within a cancer cell. In future, microarray technology will be broadly introduced into clinical practice.

## Figures and Tables

**Table 1:** Panel of possible marker genes for local tumour invasion and liver metastases in pancreatic carcinoma: function and statistical significance

**Gene symbol**	**Localisation**	**Description**	**Function**	***P*-value (*P*<10^−x^)**
RGS 4	LM/IF	Regulator of G-protein signalling 4	Signal transduction	23.669
Serpine (PAI)	LM/IF	Serine (or cysteine) proteinase inhibitor	Cell migration	23.555
GADD45A	LM/IF	Growth arrest and DNA-damage-inducible, alpha	Apoptosis, cell migration	19.428
BNIP3L	LM/IF	BCL2/adenovirus E1B 19 kDa interacting	Apoptosis	11.348
PTPN14	LM/IF	Protein tyrosine phosphatase, non-receptor type 14	Amino-acid dephosphorylation	10.808
VEGF	LM/IF	Vascular endothelial growth factor	Angiogenesis, cell migration, apoptosis	9.004
ENO2 (NSE)	IF	Enolase 2 (gamma, neuronal)	Glycolysis	8.463
HSPB1 (HSP27)	IF	Heat-shock 27-kDa protein 1	Glycolysis	7.163

IF=invasion front; LM=liver metastases; NSE=neuron-specific enolase.

**Table 2:**
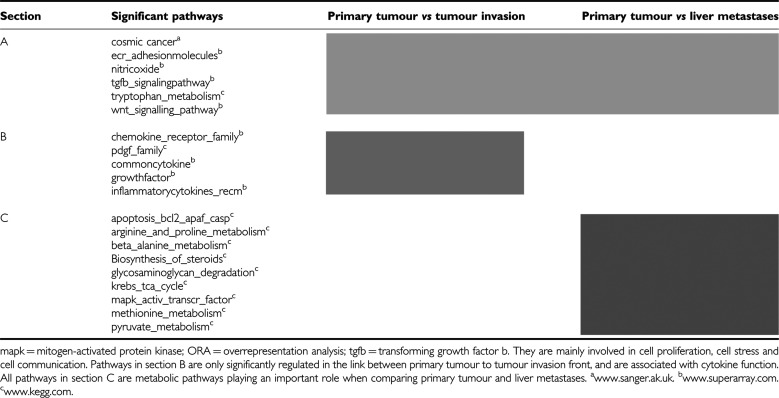
Panel of significantly regulated pathways revealed by ORA: pathways in section A are significantly regulated in both primary tumour to tumour invasion front and primary tumour to liver metastases

**Table 3:** Overview of differentially expressed genes (*P*-value<10^−*x*^) with gene symbols, *P*-values and references concerning differential expression in previous expression profiling experiments in PDAC

		***P*-value ***(10^-x^)*****			
**Gene**	**Gene symbol**	**Primary tumour *vs* tumour invasion**	**Liver metastases *vs* tumour invasion**	**Primary tumour *vs* liver metastasess**	**Reference in PDAC**
Lanyl (membrane) aminopeptidase (aminopeptidase N, aminopeptidase M, microsomal aminopeptidase, CD13, p150)	ANPEP	6.614	1.563	2.4793	1a
Actin-related protein 2/3 complex, subunit 5, 16 kDa	ARPC5	11.620	3.033	4.407	1a
BCL2/adenovirus E1B 19 kDa interacting protein 3	BNIP3L	13.809	0.273	11.348	1c
Cyclin B1	CCNB1	1.969	11.065	6.669	1b
CD24 antigen (small-cell lung carcinoma cluster 4 antigen)	CD24	6.688	3.206	0.967	1a, 1c
Cartilage oligomeric matrix protein (pseudoachondroplasia, epiphyseal dysplasia 1, multiple)	COMP	7.905	3.965	1.1798	1b
Cytochrome *c*, somatic	CYCS	6.334	6.000	0.0817	1c
Dickkopf homolog 1 (*Xenopus laevis*)	DKK1	10.739	0.074	9.286	1b
Major histocompatibility complex, class II, DQ beta 1	HLA-DQB1	6.670	2.209	1.850	1c
Immunoglobulin lambda-like polypeptide 1	IGLL1	7.157	1.889	2.466	1a
Karyopherin alpha 2 (RAG cohort 1, importin alpha 1)	KPNA2	3.197	13.764	7.706	1a
Keratin 17	KRT17	9.568	6.028	0.809	1a, 1b
Laminin, gamma 2	LAMC2	6.704	2.248	1.817	1a, 1b
met proto-oncogene (hepatocyte growth factor receptor)	MET	4.570	0.691	6.140	1b
Mesothelin	MSLN	10.732	4.173	2.637	1b
Mucin 4, tracheobronchial	MUC4	9.188	4.673	1.371	1b
Nucleosome assembly protein 1-like 1	NAP1L1	17.180	4.148	7.973	1a
Nicotinamide *N*-methyltransferase	NNMT	6.673	0.404	4.532	1b
NAD(P)H dehydrogenase, quinone 1	NQO1	18.806	5.452	8.090	1b
BH-protocadherin (brain–heart)	PCDH7	6.557	0.017	5.913	1a
Pleckstrin homology-like domain, family A, member 1	PHLDA1	3.745	2.003	7.664	1c
Prion protein (p27-30) (Creutzfeld–Jakob disease, Gerstmann–Strausler–Scheinker syndrome, fatal familial insomnia)	PRNP	9.961	0.053	8.668	1c
Protein tyrosine phosphatase type IVA, member 1	PTP4A1	4.340	1.574	7.620	1c
RAS protein activator like 2	RASAL2	6.947	3.974	0.751	1b
Solute carrier family 16 (monocarboxylic acid transporters), member 3	SLC16A3	9.039	6.121	0.571	1b
Thioredoxin reductase 1	TXNRD1	7.973	0.391	5.672	1c

PDAC, pancreatic ductal adenocarcinoma.

1a (Grützmann *et al*, 2004).

1b (Iacobuzio-Donahue *et al*, 2003b).

1c (Han *et al*, 2002).

**Table 4:** Panel of marker genes: expression values, which are standardised regarding to primary tumour and results of immunohistochemistry

	**Primary tumour**		**Invasion front**		**Liver metastases**	
	**Expression value^a^**	**IHCS**	**Expression value^a^**	**IHCS**	**Expression value^a^**	**IHCS**
VEGF	1	3	0.449	2.8	0.516	2
PAI-1	1	3	0.526	2.4	0.928	2
RGS4	1	1	0.537	1.2	0.444	0
BNIP3l	1	3	0.516	2.2	0.411	1
GADD45A	1	1	0.586	1	0.499	1
HSP27	1	3	0.670	2.6	0.561	3
PTPN14	1	2.4	0.571	2.6	0.488	1
NSE	1	3	0.568	2.4	0.590	2

GADDA45A=growth arrest and DNA damage-inducible gene 45; HSP27=heat-shock 27-kDa protein 1; IHCS=immunhistochemistry score; NSE=neuron-specific enolase; RGS4=regulator of G protein signalling 4; VEGF=vascular endothelial growth factor.

a Expression values are standardised regarding to primary tumour.
